# Colloidal chemistry with patchy silica nanoparticles

**DOI:** 10.3762/bjnano.9.278

**Published:** 2018-12-06

**Authors:** Pierre-Etienne Rouet, Cyril Chomette, Laurent Adumeau, Etienne Duguet, Serge Ravaine

**Affiliations:** 1 CNRS, Univ. Bordeaux, CRPP, UMR 5031, 115, av. du Dr Albert Schweitzer 33600 Pessac, France; 2 CNRS, Univ. Bordeaux, ICMCB, UMR 5026, av. du Dr Albert Schweitzer 33600 Pessac, France

**Keywords:** colloidal molecules, covalent bonding, patchy nanoparticles, valence

## Abstract

We report a new route to synthesize clusters, or so-called colloidal molecules (CMs), which mimic the symmetry of molecular structures made of one central atom. We couple site-specifically functionalized patchy nanoparticles, i.e., valence-endowed colloidal atoms (CAs), with complementary nanospheres through amide bonds. By analogy with the Gillespie formalism, we show that AX_4_, AX_3_E_1_ and AX_2_E_2_ CMs can be obtained from tetravalent sp^3^-like CAs when the relative amount of both building units is varied in a controlled manner. We obtain AX_2_ CMs from divalent sp-like CAs. We also show that it is possible to covalently attach two different types of satellites to the same central patchy nanoparticle to create more complex CMs, opening the way to the fabrication of new multifunctional nanostructures with well-controlled shape and composition.

## Introduction

The molecular world is essentially based on the covalent bonding of atoms displaying valences of 1, 2 (sp), 3 (sp^2^), 4 (sp^3^) and, to a lesser extent, 5 (sp^3^d) and 6 (sp^3^d^2^). The molecules of water, ammonia and methane, in which the valence orbitals of the central atom adopt sp^3^ hybridization and form equivalent bonds with two, three and four hydrogen atoms, respectively, well illustrate the great diversity of molecular structures that can be obtained. For the past two decades, this richness has been a great source of inspiration for designing colloidal analogues of molecular systems, the so-called “colloidal molecules” (CMs) [1]. Tremendous efforts have been devoted to the synthesis of particles with directional interactions to replicate bond schemes of molecular systems [2–5]. One efficient approach is to engineer particles with chemical anisotropy, that is, particles with heterogeneously surface regions in specific positions [6–12]. Bonding between particles occurs through patch–patch interactions so that the positioning of the patches can endow particles with valence. Patchy particles with various patch motifs were produced by taking benefit of the inherent directionality of colloidal clusters and by growing a matrix material onto the clusters [13–14]. By adjusting the matrix growth, it is possible to leave some zones of the clusters exposed to the outer medium, which further serve as patches with controlled size. The group of van Ravensteijn has also reported asymmetric dumbbell-like particles, i.e., with two nodules of different chemical compositions, obtained through a phase separation process during the styrene emulsion polymerization seeded with cross-linked polystyrene (PS) particles coated with a thin layer of poly(vinylbenzyl chloride) [15]. Another way to get valence-endowed particles relies on shape recognition. Shape can indeed direct colloidal assembly as it was shown that linear supracolloidal polymers are obtained by self-assembly of cone-shaped particles in the presence of a depletant [16]. Similarly, microparticles with a specific number of dimples (i.e., entropic patches [17]) can act as “locks” and assemble with small spheres (“keys”) via depletion interactions to give rise to well-defined CMs [18]. The valence of each lock particle is determined by the number of the cavities whereas their symmetry determines the bonding geometry. We have previously reported that the selective growth of the silica core of binary PS/silica CMs and the subsequent dissolution of the polymeric satellites [19] leads to silica particles with a precise number of dimples. The PS chains which are chemically grafted onto the silica surface remain at the bottom of the dimples after the dissolution stage and can be specifically chemically modified providing both enthalpic and entropic characteristics to the patches [20–21].

**Figure 1:**
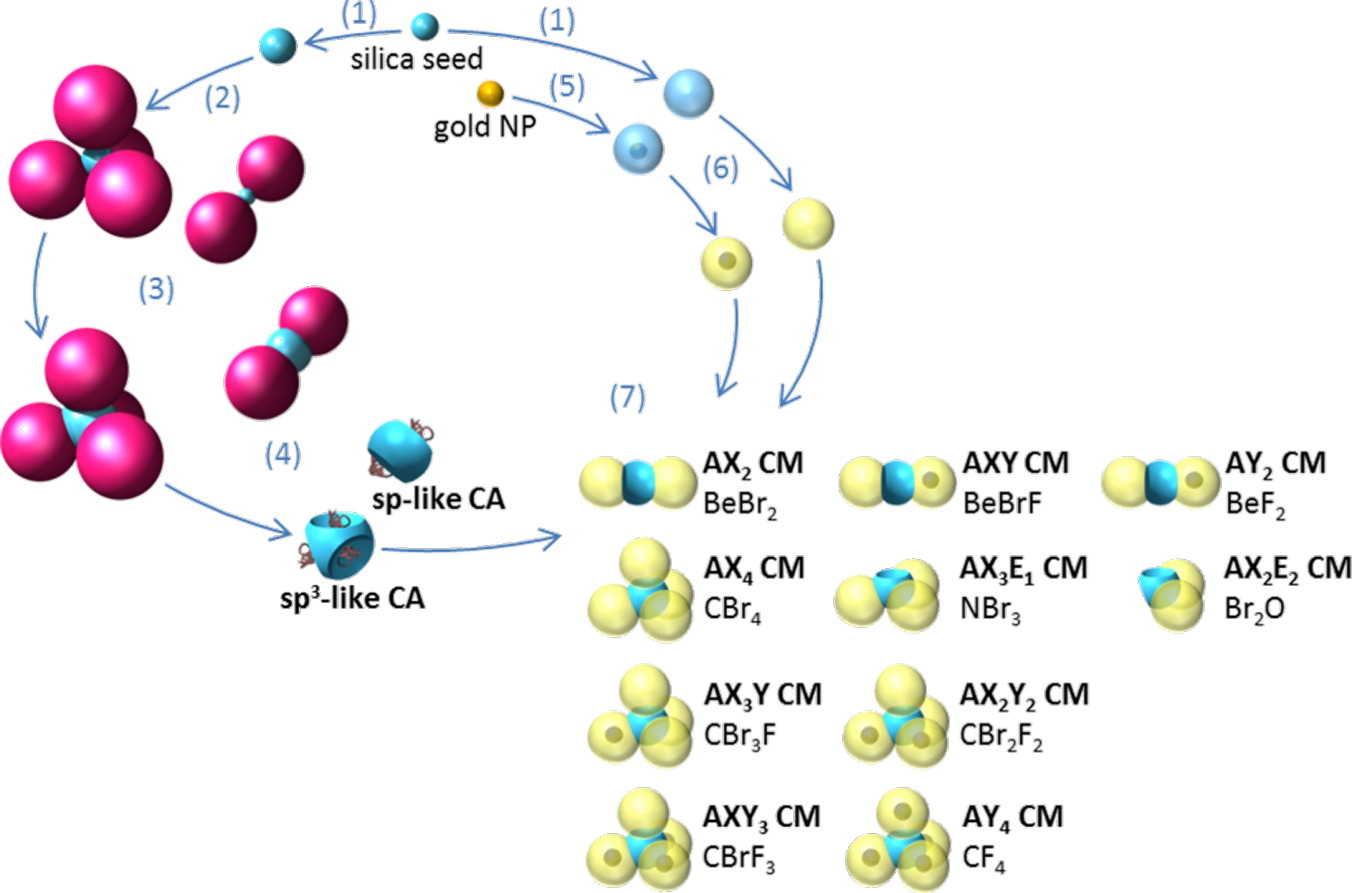
Synthesis of the CMs by covalent assembly of CAs: valence-endowed nanoparticles and nanospheres. (1) Growth of the silica seeds; (2) seeded-growth emulsion polymerization of styrene; (3) silica core regrowth; (4) dissolution of PS satellites for developing the dimples followed by the amination of the residual anchored PS chains at the bottom of the dimples; (5) growth of a silica shell around gold nanoparticles; (6) grafting of carboxylic acid groups at the silica surface of the nanospheres; (7) locking of carboxylated satellites within the aminated dimples through amide bonding.

Here we report the use of these patchy silica nanoparticles with two or four dimples as sp- and sp^3^-like colloidal atoms (CAs), respectively. We take benefit of the site-specific amination of PS residues remaining at the bottom of the dimples to form a variety of CMs through their covalent attachment with complementary spheres bearing activated carboxylic acid groups. By varying the relative amounts of both types of nanoparticles and the chemical composition of the spherical satellites, we demonstrate that a vast collection of CMs are accessible through assemblies that are analogous to chemical reactions (Figure 1).

## Results and Discussion

### Synthesis and surface modification of the precursors

**Figure 2:**
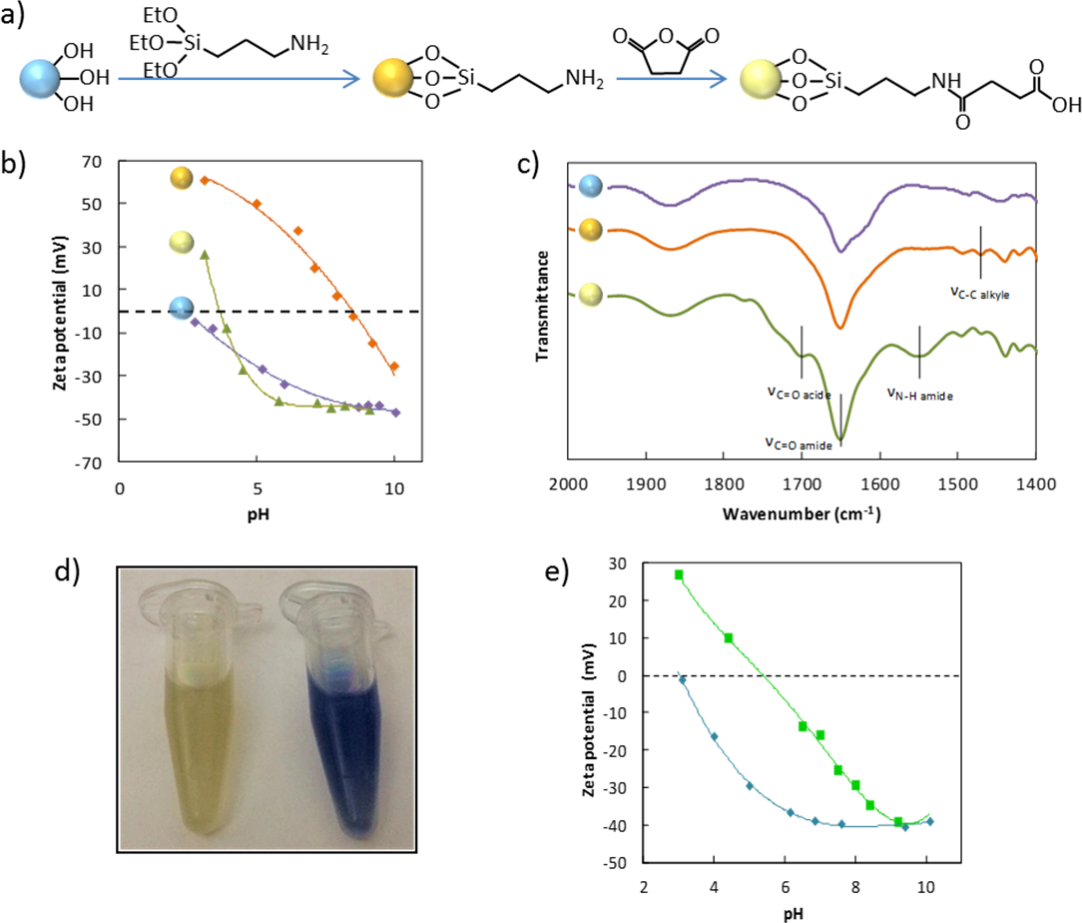
a) Silica surface modification with carboxylic acid groups, (b) zeta potential as a function of pH value, and (c) DRIFT spectra of bare (purple curve), aminated (orange curve) and carboxylated (green curve) silica particles; d) photograph of dimpled silica nanoparticles suspension before (left) and after (right) amination of the PS residues in the presence of ninhydrin; e) zeta potential as a function of pH value of silica particles with four dimples before (cyan curve) and after (green curve) chloromethylation and amination of the PS residues.

The first type of precursors, i.e., well-calibrated silica nanospheres or core–shell nanoparticles, were obtained according to a seeded-growth protocol [22] and a method using methoxy poly(ethylene glycol)-thiol as a coupling agent [23], respectively. Their surface functionalization with carboxylic acid groups was performed by a two-step approach (Figure 2a). First, amine groups were grafted onto the silica surface by reaction with (3-aminopropyl)triethoxysilane (APTES). In a second step, the amine groups were subsequently treated with succinic anhydride in the presence of triethylamine (TEA) to convert amino groups into carboxylic acid groups. The grafting efficiency was evidenced by zeta potential measurements and diffuse reflectance infrared Fourier-transform (DRIFT) spectroscopy. Figure 2b shows that after treatment of the silica surface by APTES, the so-aminated nanoparticles display a quite high zeta potential value of about 23 mV at pH 7.0. The isoelectric point (IEP) at pH 8.4 is close to the p*K*_a_ value of the primary amine groups attesting to their efficient grafting and correct orientation on the silica surface. The carboxylated particles possess a zeta potential of about −43 mV at pH 7.0 and an isoelectric point shifted down to pH 3.8, attesting to the efficient grafting of the acid groups. Figure 2c shows that the IR spectrum of the aminated particles presents a characteristic band assigned to the C–C bond of the alkyl chain of APTES groups at 1473 cm^−1^, while the IR spectrum of the carboxylated particles (Figure 2c) presents several characteristic bands such as the C=O stretching at 1712 cm^−1^ and the N–H stretching of the resulting amide at 1556 cm^−1^.

The second type of precursors, i.e., the valence-endowed patchy nanoparticles, were fabricated according to the following protocol: Bipods or tetrapods made of a central silica core and two or four PS nodules were prepared by the seeded-growth emulsion polymerization of styrene, according to an already published procedure [24]. The regrowth of the silica cores of the multipods, the subsequent dissolution of the PS nodules to create dimpled particles and the amination of the PS residues at the bottom of the dimples were performed according to a method that we had also reported previously [20]. To evidence the success of the PS modification stages, we first performed a Kaiser test, which is based on the reaction of ninhydrin with primary amines and yields a characteristic dark blue color. Briefly, 1 mL of the aminated dimpled particles suspension in absolute ethanol and 5 mg of ninhydrin were mixed in an Eppendorf tube. After few minutes, the suspension turned blue, evidencing the presence of amine functions (Figure 2d). We also measured the zeta potential of the multipods before and after the chloromethylation/amination of the PS chains (Figure 2e). The comparison of the zeta potential curves shows that the modification stages of the PS residues induce a shift of the IEP to pH 5.3 attesting to the efficient grafting of amine groups.

### Assembly of colloidal molecules

The binding of the aminated dimpled particles with the satellites is based on amide bonding, i.e., peptidic coupling, extensively studied in biochemistry for the modification of amino acids [25]. The carboxylic groups are not reactive enough toward amino groups, and a simple and well-known approach consists in converting them into more reactive groups such as anhydrides. We chose to use TEA to deprotonate the carboxylic groups and ethyl chloroformate (ECF) to react with the resulting carboxylate groups to get mixed anhydrides [26]. The assemblies were performed in dry dimethylformamide (DMF) as it is a good solvent for the aminated PS macromolecules and thus favors their extension towards the external medium, which should optimize the formation of amide bonds with the carboxylated silica satellites. At this stage, any residual water must be carefully removed to avoid the deactivation of the anhydride groups by hydrolysis.

**Figure 3:**
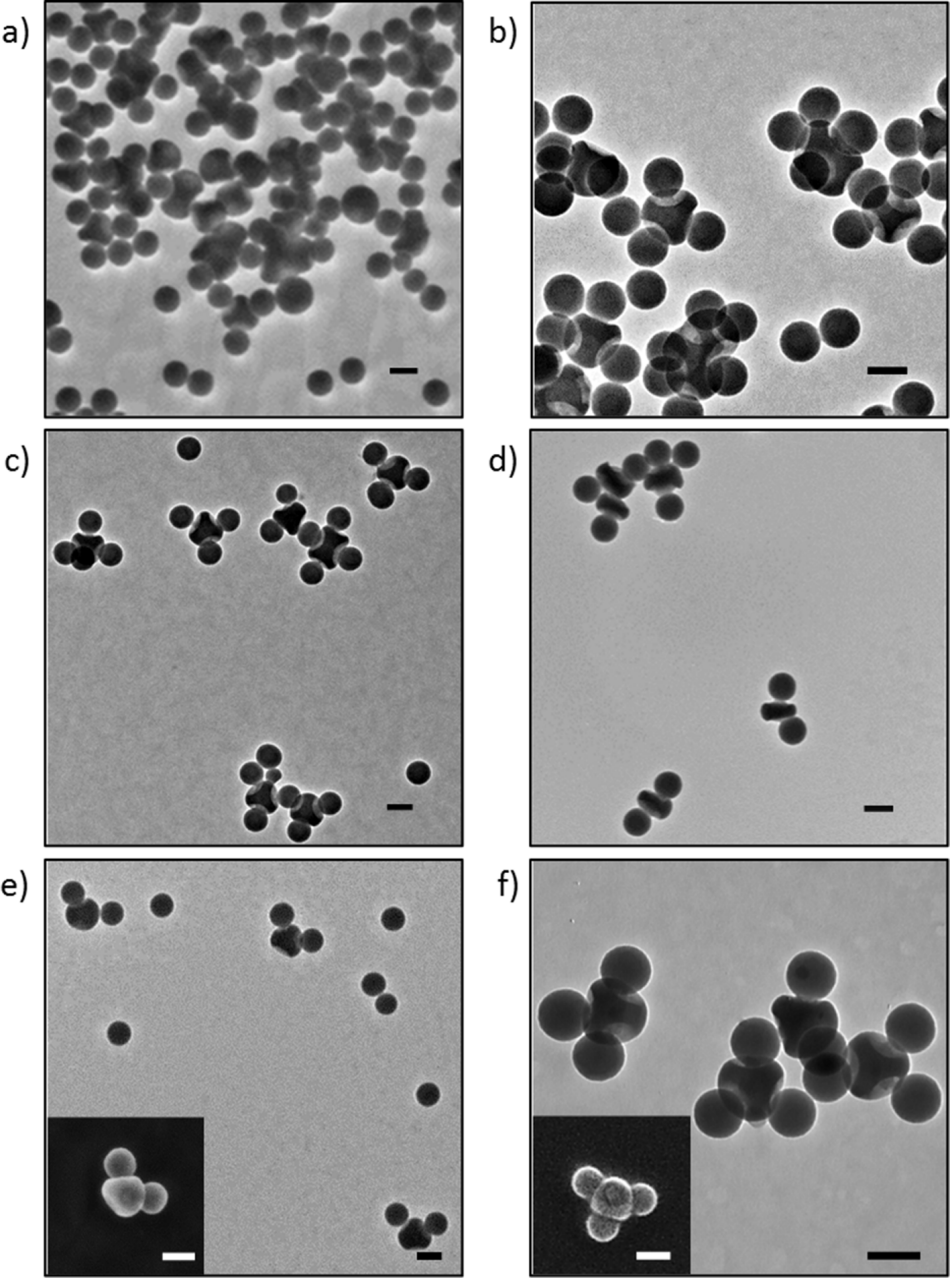
Top: TEM images of the CMs obtained from mixing particles with 4 aminated dimples with 100 nm silica nanospheres in a **1**/400 ratio before (a) and after (b) centrifugation. Middle: TEM images of the CMs obtained from mixing particles with 4 (c) and 2 (d) aminated dimples with 100 nm silica nanospheres in a **4**/4 and in a **2**/2 ratio, respectively. Bottom: TEM and SEM (inserts) images of the CMs obtained from mixing particles with 4 aminated dimples with 100 nm silica nanospheres in a **4**/2 (e) and in a **4**/3 (f) ratio. Scale bars: 100 nm.

With our collection of dimpled particles and satellites, we can build colloidal assemblies that mimic both the chemistry and the geometry of molecules. We first performed a series of experiments by mixing 100 nm carboxylated silica nanospheres with particles with four aminated dimples in a number of dimples/number of satellites ratio equal to **1**/400 in order to maximize the filling of the dimples (the bold face of the number means that it concerns the number of dimples, knowing that the number of particles is this number divided by the valency of the particle). As shown in Figure 3a, AX_4_-type CMs, the colloidal analogues of molecules such as carbon tetrabromide (CBr_4_), were obtained. One should note here that control experiments, which were carried out by mixing bare silica nanospheres with aminated dimpled CAs or carboxylated silica nanospheres with pristine dimpled CAs, both led to the observation of isolated silica nanospheres and individual CAs (not shown), evidencing that the CMs form through an amidation reaction. As expected, a large excess of free silica satellites could also be observed in Figure 3a, which was removed by centrifugation (Figure 3b). Nevertheless, the determination of the assembly yield by statistical analysis of the transmission electron microscopy (TEM) images was not possible, because CMs with a low amount of satellites could be removed with the excess of satellites during this purification step, leading distorted statistics. Therefore, we decided to perform another series of experiments dedicated to the assembly of the sp^3^-like CAs with 100 nm silica nanospheres in a **4**/4 ratio. Figure 3c shows that colloidal analogues of CBr_4_ molecules were mostly obtained, with a yield of 59% as determined by statistical analysis of TEM images (Table 1). Similarly, AX_2_-type CMs, analogues of molecules like beryllium bromide (BeBr_2_) were obtained (Figure 3d) , with a yield of 62% (Table 1) when sp-like CAs were mixed with 100 nm silica nanospheres in a **2**/2 ratio.

**Table 1:** Compositions of the batches^a^ resulting from mixing the CAs with 100 nm nanospheres.

	sp^3^-like CAs			sp-like CAs
number of dimples / number of nanospheres	**4**/4^b^	**4**/3^c^	**4**/2^d^	**2**/2^e^

% AX_4_-type CMs	59	20	5	—
% AX_3_E_1_-type CMs	25	58	27	—
% AX_2_E_2_-type CMs	15	19	35	—
% AXE_3_-type CMs	1	3	22	—
% AX_2_-type CMs	—	—	—	62
% AX_1_E_1_-type CMs	—	—	—	29
% unreacted CAs	—	—	11	9

^a^Determined by statistical analysis of TEM images over ^b^140, ^c^200, ^d^116, and ^e^182 clusters.

Based on these results, we decided to extend our synthetic approach in order to first reproduce at the colloidal level the fact that a central atom can be surrounded by both bonded atoms and lone electron pairs. To do so, we decided to change the quantity ratio of both types of precursors. Hence, we mixed the sp^3^-like CAs with 100 nm silica nanospheres in ratios of **4**/2 and **4**/3. Figure 3e,f show that AX_2_E_2_-type and AX_3_E_1_-type CMs, which are the colloidal analogues of molecules such as dibromine monoxide (Br_2_O) and nitrogen tribromide (NBr_3_), were obtained with a good yield (Table 1), respectively. In particular, the unfilled dimple(s) of the AX_2_E_2_- and AX_3_E_1_-type CMs can be clearly seen on the scanning electron microscopy (SEM) images shown as inserts of Figure 3e,f.

**Figure 4:**
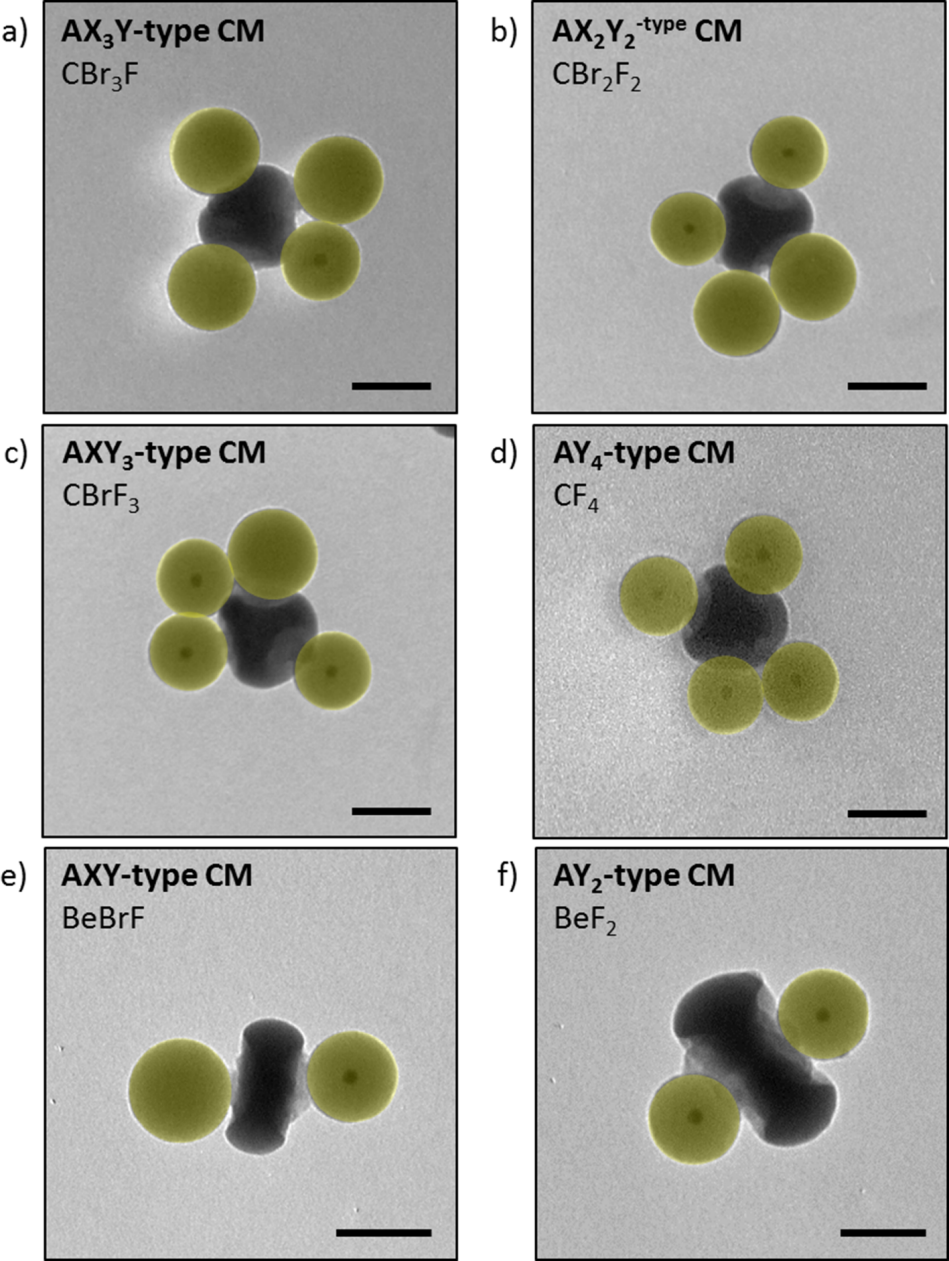
TEM images of the CMs obtained from mixing particles with four aminated dimples with 100 nm silica nanospheres and 90 nm core–shell nanoparticles in ratios of (a) **4**/3/1, (b) **4**/2/2, (c) **4**/1/3 and (d) **4**/0/4. TEM images of the CMs obtained from mixing particles with two aminated dimples with 100 nm silica nanospheres and 90 nm core–shell nanoparticles in ratios of (e) **2**/1/1 and (f) **2**/0/2 ratio; scale bars: 100 nm.

We also aimed to mimic at the colloidal scale the possible bonding of atoms of different natures to a same central atom, which is the source of the richness of the organic molecules. We focused on AX*_n_*Y_4−_*_n_*-type and AX*_n_*Y_2−_*_n_*-type CMs where 0 < *n* < 4 and 0 < *n* < 2, respectively. We used 90 nm core–shell nanoparticles as a second batch of satellites. This choice was motivated by two reasons. Firstly, we had to work with satellites with a slightly different diameter to mimic another type of atoms without modifying the valence of the dimpled particles. By doing so, we were sure to attach only one satellite per dimple, whatever its nature. Using much smaller satellites could induce the attachment of more than one satellite per dimple. In contrast, the dimples of the patchy particles must be big enough to allow one satellite to be linked to the aminated PS chains, which excludes to work with too large satellites. The second reason is related to the necessity to differentiate both types of satellites attached to a central dimpled particle by conventional TEM. Thanks to the high electron density of gold, core–shell nanoparticles can indeed be easily distinguished from silica nanospheres of similar size. We mixed the sp^3^-like CAs with 100 nm nanospheres and 90 nm core–shell nanoparticles in a **4**/*n*/(4 − *n*) ratio. Figure 4a–d shows that the colloidal analogues of molecules such as fluorotribromomethane (CFBr_3_), difluorodibromomethane (CF_2_Br_2_), bromotrifluoromethane (CF_3_Br), and carbon tetrafluoride (CF_4_) are formed with a yield equal to 41%, 50%, 43% and 52 %, for *n* = 3, 2, 1 and 0, respectively. Similarly, the analogues of molecules such as beryllium fluoride bromide (BeFBr) and beryllium fluoride (BeF_2_) were obtained from sp-like CAs in a **2**/1/1 and in a **2**/0/2 ratio, with a yield of 49% and 54%, respectively (Figure 4e,f).

## Conclusion

In conclusion, colloidal molecules, which mimic the symmetry of molecular structures, have been synthesized through the covalent attachment of spherical satellites within the dimples of valence-endowed patchy nanoparticles. The synthetic route is highly versatile and can be extended to other precursors such as metallic or semiconductor nanoparticles, opening the way to the synthesis of a broad panel of (multi)functional nanomaterials with a controlled shape and composition. These new colloidal analogues of molecules could serve as building blocks for the assembly of the next generation (meta-)materials. For example, attaching four different satellites (such as one gold, one silver, one iron oxide and one semiconductor nanoparticle) around a central sp^3^-like nanoparticle could lead to multifunctional chiral nanostructures, which may form novel two- or three-dimensional materials with unprecedented properties by self-assembly.

## Experimental

### Materials

We used styrene (Sigma-Aldrich, 99%), methacryloxymethyltriethoxysilane (MMS, ABCR, 98%), methacryloxypropylyltriethoxysilane (MPS, Aldrich, 98%), (3-aminopropyl)triethoxysilane (APTES, Aldrich, 99%), triethylamine (TEA, Sigma-Aldrich, 99%), sodium persulfate (Sigma-Aldrich, 99%), Symperonic^®^ NP30 (Aldrich), sodium dodecylsulfate (SDS, Sigma-Aldrich, >90%), tetraethoxysilane (TEOS, Sigma-Aldrich, 99%), ammonia (30% in water, SDS), tin tetrachloride (SnCl_4_, Sigma-Aldrich, >99%), hydrochloric acid (37%, Sigma-Aldrich), ethylenediamine (Fluka, 99.5%), gold(III) chloride trihydrate (HAuCl_4_·3H_2_O, Sigma-Aldrich), trisodium citrate dihydrate (NaCit) (Na_3_C_6_H_5_O_7_·2H_2_O, Sigma-Aldrich, 99%), *O*-[2-(3-mercaptopropionylamino)ethyl]-O′-methylpoly(ethylene glycol) (PEG-SH, *M*_w_ = 5000) as we received them. We systematically used ultrapure water at 25 °C obtained from a Milli-Q system (Millipore). We purchased tetrahydrofuran (THF), dimethylformamide (DMF) from Sigma-Aldrich and chloroform and absolute ethanol from VWR Chemicals. Butyl chloromethyl ether was synthesized according to a recipe already published [27].

### Synthesis and surface modification of the spherical satellites

#### Synthesis of the “pre-seeds”

In a similar manner as described in [28], 100 mL of L-arginine aqueous solution (6 mM) were added into a 150 mL vial thermostated with hot water circulation at 60 °C and equipped with a reflux condenser and a 3 cm Teflon^®^-coated stirring bar. When a constant temperature of 60 °C was reached, 10 mL of TEOS were gently added in order to create a top organic phase. The stirring rate was adjusted in order to maintain the organic phase undisturbed and the aqueous phase efficiently mixed (ca. 150 rpm). The reaction was stopped after three days. Silica concentrations were determined by gravimetric analysis. In a given volume, the number of silica seeds was calculated from the silica concentration and the particle average diameter (ca. 23 nm) and assuming that the particles were spherical and their density was 2.2 g·cm^−3^.

#### Synthesis of the silica nanospheres

Regrowth stages were performed according to a previously reported protocol [28] at room temperature in a conventional glass flask where 455 mL of ethanol, 35 mL of ammonium hydroxide and 10 mL of the aqueous dispersion of silica “pre-seeds” were successively introduced. Then, a calculated amount of TEOS (Table 2) was added at the rate of 0.5 mL·h^−1^. The mixture was stirred until 2 h after the end of the TEOS addition.

**Table 2:** Experimental conditions of the silica particles synthesis and size measurement results of the silica particles obtained (as extracted from statistical analysis of TEM images).

TEOS/silica (weight ratio)	*D*_silica_ (nm)	PDI

28	48	1.042
33	53	1.035
218	103	1.027

The polydispersity index (PDI) given in Table 2 was calculated based on a minimum of 500 nanoparticles per batch using the following equation:


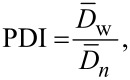


where


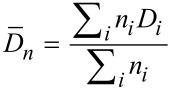


and


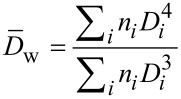


are the number-average and the weight-average diameter, respectively, and *n_i_* is the number of particles of diameter *D_i_.*

#### Synthesis of the Au@SiO_2_ nanoparticles

Gold nanoparticles of 14 ± 2 nm were prepared by the citrate-reduction method reported by Turkevich [29]. SiO_2_ coating was carried out after surface functionalization of the gold nanoparticles by using a PEG-SH (*M*_w_ = 5000) aqueous solution in a similar manner as described in [23]. The surface modification allowed for the replacement of the citrate molecules adsorbed onto the gold surface by PEG-SH. A ratio of four PEG molecules per square nanometer of available surface of the gold sol was fixed. The aqueous solution of PEG-SH was freshly prepared and added dropwise to the as-prepared gold nanoparticles under vigorous magnetic stirring. The mixture was left to react for 2 h, and was centrifuged at 9000 rpm for 30 min (twice) in order to eliminate undesired reactants. The AuNPs@PEG-SH nanoparticles were redispersed in absolute ethanol. To coat the gold nanoparticles with a silica shell, 15 mL of the Au nanoparticles dispersion were mixed under continuous magnetic stirring with a solution of deionized water and ammonia, at a volume ratio of 93.8/5/1.2 for absolute ethanol, water and ammonia, respectively. 18.3 μL of TEOS were added and the reaction mixture was stirred for 12 h at 20 ± 2 °C. Upon completion of the growth of silica shell, the core–shell particles were washed with absolute ethanol and water and redispersed in absolute ethanol.

#### Grafting of carboxylic acid groups onto the surface of the spherical satellites

We quickly added under vigorous stirring a pre-determined volume of APTES, corresponding to a nominal surface density of 20 functions per square nanometer, into the suspension of the as-prepared silica nanoparticles. The mixture was stirred at room temperature for 12 h to promote covalent bonding. The particle suspension was purified by three cycles of centrifugation/redispersion (10,000*g*; 20 min) in absolute ethanol. Then, a given volume of TEA corresponding to a nominal surface density of 50 functions square nanometer was added into the particle suspension. The mixture was stirred at 60 °C for 15 h before being centrifuged (10,000*g*; 20 min). The particles were redispersed in 30 mL of anhydrous DMF and a given volume of succinic anhydride corresponding to a nominal surface density of 50 functions square nanometer was added. The mixture was stirred at 60 °C for 15 h to promote covalent bonding. The particle dispersion was purified by two cycles of centrifugation (12,000*g*; 10 min)/redispersion in absolute ethanol followed by two cycles of centrifugation (12,000*g*; 10 min)/redispersion in anhydrous DMF.

### Synthesis of the dimpled silica particles with aminated PS chains

#### Synthesis of the multipod-like PS/silica clusters

We prepared batches of bipods and tetrapods, consisting of a central silica core surrounded by two or four PS satellite nodules, by seeded-growth emulsion polymerization of styrene, according to a procedure we published previously [24]. We used two batches of silica seeds with diameters of 48 and 53 nm, respectively, previously surface-modified with MMS or MPS (0.5 molecules per square nanometer) and a surfactant mixture (3 g·L^−1^) of Symperonic^®^ NP30 and SDS. The polymerization was performed at 70 °C for 6 h.

#### Regrowth of the silica core

We regrew the silica cores to create dimples using a method that we had reported previously [19]. We prepared a mixture of 227.5 mL ethanol and 17.5 mL ammonia (1 M) and added first 5 mL of the polymerization medium containing clusters and a given volume of TEOS at a rate of 1 mL·h^−1^. The experimental conditions and composition of the batches are described in Table 3. For dissolving the PS satellites, we added a volume of DMF corresponding to 10% of the total volume. Then, the dispersion was heated at 70 °C and partially evaporated under vacuum using a rotary evaporator. Then, the temperature was increased to 90 °C and the evaporation continued until the dispersion turns from white to almost transparent. The removal of the dissolved PS was performed by three cycles of centrifugation (10,000*g*; 10 min) and redispersion in THF.

**Table 3:** Experimental conditions and final compositions of the multipod-like silica/PS clusters used in this study and their geometrical features.

		bipod batch	tetrapod batch

experimental conditions for the seeded-growth emulsion polymerization of styrene	*D*_silica_ (nm)	48	53
	*N*_silica_ (10^15^ L^−1^)	18	18
	*S*_silica_ (m^2^·L^−1^)	130	159
	[styrene]_0_ (g·L^−1^)	100	100
	coupling agent	MPS	MMS
	NP30/NP30 (%/%)	98/2	95/5
	styrene-to-PS conversion (%)	70	84

experimental conditions for the silica core regrowth	[clusters] (10^14^ NPs·L^−1^)	3.6	3.6
	added *V*_TEOS 10 % in ethanol_ (mL)	5	5
	% bipods	86	4
	% tripods	11	19
	% tetrapods	1	76
	% others	2	1
	*D*_silica_ after regrowth (nm)	131	132

#### Amination of the residual PS macromolecules at the bottom of the dimples

We used a recipe already reported [21]. Briefly, after transferring the as-prepared dimpled silica particles in chloroform, we added butyl chloromethyl ether in chloroform (3 M; 5 mL) and 0.3 mL SnCl_4_. We set the temperature to 45 °C and then aged the mixture overnight. Finally we washed the nanoparticles by three cycles of centrifugation (5000*g*; 15 min) and redispersion in HCl aqueous solution (4 wt %) and then in water/ethanol (50/50 wt/wt) before redispersion in 20 mL DMF. We performed the amination stage by using 10^13^ chloromethylated patchy particles in DMF and 3 mL ethylenediamine, i.e., in large excess for minimizing cross-linking. We set the temperature at 90 °C and let the system react overnight under stirring. We washed the nanoparticles by two cycles of centrifugation (12,000*g*; 20 min) and redispersion in ethanol and two extra cycles in water. After having made the amino groups protonated using few drops of HCl, we centrifuged the dispersion and redispersed the nanoparticles in DMF.

#### Assembly of the aminated dimpled particles with the carboxylated silica spheres

The carboxylic acid functions at the surface of the silica spheres were transformed into carboxylic anhydrides through the addition of ECF. To do so, 12 μL of TEA (which correspond to a surface density of 8 functions per square nanometer) were added into 5 mL of a suspension of particles in anhydrous DMF (at 46 g·L^−1^). After homogenization 4 μL of ECF were added. The suspension was stored at 4 °C. A given volume of the activated silica spheres was mixed with the aminated dimpled particles in an Eppendorf tube and the mixture was gently shaken for one week. The clusters were collected by three cycles of centrifugation (500*g*; 20 min) and redispersion in ethanol.

### Characterization techniques

#### Transmission electron microscopy (TEM)

TEM experiments were performed using a Hitachi H600 microscope operating at an acceleration voltage of 75 kV and a JEOL JEM 1400 Plus microscope operating at 120 kV. We prepared the samples by depositing one drop of the colloidal dispersion on conventional carbon-coated copper grids. We let the liquid evaporate in the open air at room temperature and placed the grids in a box away from dust.

#### Scanning electron microscopy (SEM)

We performed high-resolution SEM experiments with a JSM 6700F microscope at the Plateforme de Caractérisation des Matériaux d’Aquitaine (PLACAMAT). One drop of the CMs suspension was deposited on a glass slide. It was then dried in the open air, metalized, and placed in a box away from dust.

#### Diffuse reflectance infrared Fourier-transform (DRIFT) spectroscopy

We evaporated solvent from the solution of PS or modified PS. To 9 mg of the dried sample we added 281 mg of desiccated KBr (spectroscopy grade). We ground the mixture in an agate mortar and deposited the powder on the sample holder. The sample was then introduced into the Bruker IFS Equinox 55 spectrometer and the measurements were performed in a Selector Graseby Specac reflection cell. After 30 min of degassing, the infrared spectrum was recorded by the acquisition of 120 measurements with a resolution of 2 cm^−1^.

#### Zeta potential measurements

The nanoparticle dispersion was diluted to a concentration of about 10^15^ NPs·L^−1^. The pH value of the solution was adjusted by the addition of HCl (0.1 M) or NaOH (0.1 M). When the desired pH value was reached, a volume of 5 mL of the sample was collected. After equilibration of the pH value for 12 h, the pH value of the samples was measured once again and the zeta potential values were measured using the Malvern Zetasizer 3000 HS setup (Malvern Instruments). Each measurement was performed for 30 s, the dielectric constant of solvent (water) was set to 80.4 and the Smoluchowsky factor *f*(κa) was 1.5.
